# RET Gene Fusions in Malignancies of the Thyroid and Other Tissues

**DOI:** 10.3390/genes11040424

**Published:** 2020-04-15

**Authors:** Massimo Santoro, Marialuisa Moccia, Giorgia Federico, Francesca Carlomagno

**Affiliations:** 1 Department of Molecular Medicine and Medical Biotechnology, University of Naples "Federico II", 80131 Naples, Italy; lisa.moccia@hotmail.it (M.M.); giorgia.federico@libero.it (G.F.); francesca.carlomagno@unina.it (F.C.); 2 Institute of Endocrinology and Experimental Oncology of the CNR, 80131 Naples, Italy

**Keywords:** kinase, tyrosine kinase inhibitor, targeted therapy, thyroid cancer

## Abstract

Following the identification of the BCR-ABL1 (Breakpoint Cluster Region-ABelson murine Leukemia) fusion in chronic myelogenous leukemia, gene fusions generating chimeric oncoproteins have been recognized as common genomic structural variations in human malignancies. This is, in particular, a frequent mechanism in the oncogenic conversion of protein kinases. Gene fusion was the first mechanism identified for the oncogenic activation of the receptor tyrosine kinase RET (REarranged during Transfection), initially discovered in papillary thyroid carcinoma (PTC). More recently, the advent of highly sensitive massive parallel (next generation sequencing, NGS) sequencing of tumor DNA or cell-free (cfDNA) circulating tumor DNA, allowed for the detection of RET fusions in many other solid and hematopoietic malignancies. This review summarizes the role of RET fusions in the pathogenesis of human cancer.

## 1. The RET Receptor 

RET (REarranged during Transfection) was initially isolated as a rearranged oncoprotein upon the transfection of a human lymphoma DNA [1]. The *RET* gene maps on human chromosome 10 (at q11.21) [2] and codes for the functional tyrosine-kinase receptor (RTK) of GDNF (glial cell line-derived neurotrophic factor), Neurturin (NRT), Artemin (ART), and Persephin (PSF) growth factors [3,4,5,6,7]. These growth factors bind to auxiliary membrane-bound co-receptors, named GFR- s (GDNF family receptor- [1,2,3,4]), thereby forming a bipartite complex that, in turn, mediates RET dimerization and activation [8]. In humans, mutations of this ligand-receptor system cause intestinal aganglionosis with congenital megacolon (Hirschsprung disease) and congenital defects of kidney and urinary tract [9]. 

**Figure 1:**
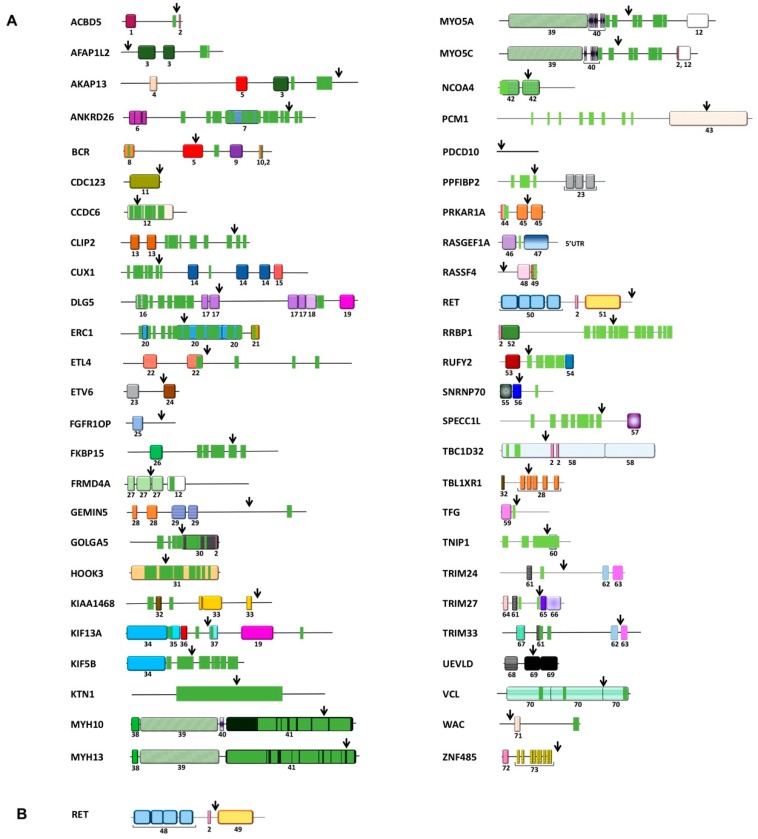
Representative scheme of RET and its fusion partners. (**A**) Representation of RET fusion protein partners. Arrows indicate the most frequent breakpoint sites in partner proteins. The number under each protein domain refers to the protein domain legend (Table 1). Coiled-coil domains are very numerous and, therefore, are represented as light green boxes without number. (**B**) Representation of the RET protein. Arrow indicates the most frequent breakpoint site in RET.

Structurally, the RET protein is composed by an extracellular (EC), a transmembrane (TM), and an intracellular (IC) portion (Figure 1). RET-EC contains 4 cadherin-like (CLD) and one cysteine-rich (CRD) domains, that are involved in binding to the bipartite ligand [8]. RET-IC contains the tyrosine kinase domain (TKD) that is split into two subdomains [7,10]. This is followed by a C-terminal tail that is subject to alternative splicing generating different isoforms, the most abundant being RET9 and RET51 (depending whether they contain 9 or 51 residues starting from glycine 1063 in exon 19) [3]. 

Upon activation, several tyrosine residues of RET-IC undergo phosphorylation and mediate intracellular signal transduction. Thus, tyrosines Y900, Y905, Y981, Y1015, Y1062, and Y1096 (this one is specific for RET51) have been involved in functional RET signaling. Phosphorylated tyrosine 1062 (Y1062), in particular, recruits a multitude of adaptors such as SHC1/3, FRS2, IRS1/2, and DOK1/4/5 that, in turn, mediate the activation of RAS (Rat Sarcoma)-MAPK (Mitogen-Activated Protein Kinases) and PI3K (Phosphatidylinositol-3 Kinase)-AKT (Protein Kinase B) pathways [3,4,5,6,7].

## 2. *RET* Oncogenic Conversion in Human Neoplasms

Different *RET* molecular lesions have been described in tumors at either germline or somatic levels. These include gene amplification, fusion, as well as single base substitutions/small insertions/deletions either in sequences encoding RET-EC or -IC. Germline or somatic single base substitutions/small insertions/deletions in *RET* are characteristic of sporadic or familial (MEN2—multiple endocrine neoplasia type 2 associated) medullary thyroid carcinoma (MTC), respectively. Instead, *RET* fusions, occurring at the somatic level, are typical of papillary thyroid carcinoma, lung adenocarcinoma, and few other cancers. This notion has made RET an attractive molecular target for small molecule tyrosine kinase inhibitors (TKI) [11,12,13,14]. In this frame, novel selective RET TKIs have featured promising results in clinical investigation [14,15].

For a fully comprehensive description of the role played by RET in cancer, the reader is referred to other Reviews published on the topic (see References [4,5,6,7,16,17,18]). Moreover, comprehensive annotation of *RET* genetic lesions in cancer are provided by TCGA PanCancer, AACR GENIE, and MSKCC projects [19,20,21].

This review addresses, in particular, the role of *RET* gene fusions in cancer. Table 1 lists *RET* fusions so far described and Figure 1 depicts the protein structure of RET and its fusion partners.

## 3. Functional Consequence of *RET* Gene Fusions

RTK fusions in cancer may either result in the juxtaposition of a N-terminal partner to the C-terminal portion of the RTK, including its catalytic domain (so called 3’ kinase fusion), or, vice versa, of the N-terminal portion of the RTK, with its catalytic domain, to the C-terminal of a fusion partner (5’ kinase fusion) [77]. In both cases, the retention of an intact kinase domain in the fusion product is essential to distinguish cancer-driving RTK fusions from random chimeric products secondary to genetic instability [77]. 3’ kinase fusions are the most common; however, examples of 5’ kinase fusions have also been described, such as FGFR2 and FGFR3 (fibroblast growth factor receptors) fusions in cholangiocarcinoma and other malignancies [78] as well as the EGFR (epidermal growth factor receptor)-RAD51 (Radiation-sensitive 51) and -PURB (Purin Rich Beta) fusions in lung cancer [79]. One argument supporting the selection of 3’ kinase fusions in cancer may be that this type of fusion is able to move the kinase domain under the control of the transcriptional promoter of the fusion partners, thus fostering aberrant kinase overexpression (see also below) [77]. The cancer-associated *RET* fusions described so far are of the 3’ kinase fusion type, involving a 5’-terminal partner coding sequence fused to the 3’-terminal *RET* kinase domain coding sequence. Breakpoints in *RET* and its fusion partners typically occur in intronic regions and are able to preserve, upon mRNA splicing, an open reading frame. Most commonly, secondary to a breakpoint in *RET* intron 11, the *RET* coding sequence from exon 12 to the STOP codon is included in the fusion. However, in rare instances, the fusion product starts from *RET* alternative exons, such as exon 3, 7, 9 (EC portion), 10 (TM: transmembrane segment), or 11 (IC portion). 

*RET* gene fusions lead to kinase activation, in turn augmenting signal transduction along classical pathways such as the MAPK and the PI3K/AKT ones [4,5,80]. This causes gain of RET oncogenic activity, as shown for the most frequent fusions (CCDC6-RET, NCOA4-RET, KIF5B-RET) using cell-based assays as well as constitutive or conditional transgenic mouse models [28,29,50,51,81,82,83,84]. Various mechanisms may contribute to fusion-mediated RET activation, including: (i) as mentioned above, the increased kinase expression, due to the replacement of the 5’-upstream *RET* promoter, that is normally active mainly in neuronal cell lineages, with that of the fusion partners [85], and (ii) the dimerization/oligomerization of the RET kinase domain mediated by protein–protein interaction motifs, typically coiled-coil domains, present in the fusion partners, that leads to ligand-independent kinase activation [65,86]. In addition, loss of autoinhibitory N-terminal portions and the altered conformation of the rearranged kinase may also contribute to RET kinase activation [87]. 

Besides the aberrant expression and the gain of intrinsic RET kinase activity, it is possible that other mechanisms contribute to the oncogenic role of RET fusions. First, the altered intracellular localization, due to the loss of the RET signal peptide and TM, may participate to RET transforming ability, for example by facilitating rearranged RET coupling to intracellular signal transducers. In addition, the RET fusion partner may act as a protein–protein interaction platform able to modify RET kinase-substrate(s) binding. Accordingly, in the KIF5B (Kinesin-1 heavy chain)-RET fusion, commonly found in lung carcinoma (see below), the N-terminal KIF5B backbone, containing the kinesin motor domain, was demonstrated to activate, through RAB GTPase vesicles, multiple RTKs, including EGFR and FGFR, and their downstream signaling [88]. Furthermore, the interaction of the chimeric RET kinase with another RTK may potentiate signaling and reduce efficacy of RET-targeted TKIs. In this frame, it has been shown that the physical interaction between CCDC6-RET and the EGFR facilitates signaling and reduces efficacy of RET kinase inhibition in a lung cancer cell line [89]. Finally, the concurrent altered function of the RET fusion partner may also contribute to neoplastic transformation. Accordingly, several RET fusion partners have been shown to exert relevant homeostatic functions (Table 1). PRKAR1A, one of the RET fusion partners, is the tumor suppressor protein involved in inheritance of Carney complex, a tumor-prone syndrome [90]. Similarly, another RET fusion partner, NCOA4, is a multifunctional protein that is involved in the regulation of DNA synthesis by inhibiting replication origin activation. Alteration of such a function in tumors harboring the NCOA4-RET fusion may contribute to its oncogenic activity [91]. Finally, CCDC6 exerts proapoptotic and DNA damage response activities and perturbation of these functions may participate to the oncogenic role of the corresponding RET fusion [92].

## 4. Genomic Mechanism of *RET* Gene Fusions

Different inter-chromosomal (translocations) or intra-chromosomal (inversions, tandem duplications, and interstitial deletions) structural variations can result in gene fusion events [87,93]. As discussed below, the most common RET fusions, in papillary thyroid carcinoma (PTC) and in lung adenocarcinoma (LADC) are CCDC6-RET and NCOA4-RET (primarily in PTC) and KIF5B-RET (primarily in LADC). *CCDC6* and *NCOA4* genes both map on the long arm of chromosome 10, at 10q21.2 or 10q11.22, respectively (Table 1). *RET* (that maps at 10q11.21) is transcribed in an opposite orientation with respect to *CCDC6* and *NCOA4*. Thus, a 10q paracentric (not including the centromere, e.g., with both breakpoints in the same chromosome arm) inversion is the plausible genomic mechanism underlying CCDC6-RET and NCOA4-RET fusions [94]. Instead, *KIF5B* maps at 10p11.22; therefore, a pericentric (including the centromere, e.g., with a breakpoint in each chromosome arm) inversion of chromosome 10 is the mechanism of KIF5B-RET fusion (Table 1) [29,50,51,95]. In principle, inversions may be reciprocal (both fusion products) or nonreciprocal (only one fusion product). While in PTC, the majority of the inversions were reciprocal, in LADC, nonreciprocal inversions have also been documented [95].

*RET* gene fusions are thought to occur secondary to DNA double-strand breaks (DSB) followed by illegitimate repair of the two unrelated genes [13,18,95,96]. DNA repair mechanisms that can be responsible include NHEJ (nonhomologous end joining), which requires no homology or just very short homology sequences and inserts a few nucleotides at the fusion point, and BIR (break-induced replication), that instead requires long homology DNA stretches [95,96]. Sequencing of *RET* breakpoints in LADC suggested that both mechanisms can be involved, while, at least in radiation-associated PTC, NHEJ was the prevalent one [95,97]. 

In turn, DSBs can be induced by endogenous sources of DNA damage, such as reactive oxygen species (ROS) and replication stress, or exogenous sources, such as ionizing radiation (IR), see also below [98,99]. Chromosomal fragile sites are DSB-prone genome regions, in particular, under conditions that reduce DNA replication or upon the exposure to several chemical agents. It is worth noting that *RET* and *NCOA4* are located within the fragile site FRA10G, and *CCDC6* is located within the fragile site FRA10C, a fact that may facilitate their rearrangement [100,101,102,103]. Moreover, breakpoints in *RET* intron 11 map close to DNA topoisomerase 1/2 predicted cleavage sites, suggesting that DNA topoisomerases may play a role in *RET* fusion events [102]. Accordingly, during replication and transcription, DNA is unwound by DNA helicase, resulting in torsion that can be removed by topoisomerases through transient introduction of DNA breakage, thus potentially favoring gene rearrangements in cancer cells [102]. Finally, tissue-specific organization of chromatin territories may affect the frequency as well as the specificity of *RET* fusion with specific gene partners. In this frame, the overlap in thyrocyte interphase chromatin of *CCDC6*, *NCOA4*, and *RET* loci is important to favor their fusion [100].

Chromoplexy occurs when several DSBs are generated at the same time in different chromosomes. This is followed by chains of rearrangements, resulting in the simultaneous shuffling of several chromosomal fragments. Chromoplexy is typically found in prostate carcinoma and lymphoid malignancies [93]. However, in the recent pan-cancer analysis of whole genomes, it was found that 4 of the 13 fusion genes identified in thyroid carcinoma, including 2 *RET* fusions, were caused by chromoplexy [93].

## 5. *RET* Gene Fusions in Thyroid Carcinoma

Papillary thyroid carcinoma (PTC) is the most common (~80%) malignancy of the thyroid gland. PTC was the first human cancer to be consistently associated to RET fusions (so called RET/PTC rearrangements) (Table 1) [28,104]. Overall, gene fusion is a common genomic structural variation in PTC, targeting, besides RET, several other kinases, such as NTRK1, NTRK3 (N Tropomyosin Receptor Kinase), ALK (Anaplastic Lymphoma Kinase), HGFR (Hepatocyte Growth Factor Receptor), and BRAF (B Rapidly Accelerated Fibrosarcoma) [19,77]. 

The most common RET fusions in PTC are CCDC6-RET (also named RET/PTC1) and NCOA4-RET (also named RET/PTC3), accounting for about 90% of RET fusion-positive cases [80]. CCDC6 (coiled-coil domain containing 6) was formerly known as H4 or D10S170 and NCOA4 (nuclear coactivator 4) was formerly known as ELE1, RFG, or ARA70 (Table 1). Although alternative breakpoints have been described, the breakpoint cluster region typically maps in *RET* intron 11, in *CCDC6* intron 1, and in *NCOA4* intron 8 (Figure 1) [24].

PTC is typically associated with lesions in genes causing unscheduled activation of the MAPK (mitogen-activated protein kinase) cascade. Indeed, besides RET and other RTKs, common PTC-driver events are represented by gain-of-function mutations of RAS small GTPases, and, most commonly, BRAF kinase. The mutually exclusive nature of these lesions supports the notion they act along a common signaling mechanism [104]. In the TCGA study, RET-, similarly to BRAF-positive samples, were “classic” papillary cases, whereas RAS-positive samples typically featured the follicular variant phenotype [24].

RET fusions occur in ~10%–20% of sporadic PTCs [15,18]. In the TCGA study, enrolling almost 500 PTC samples, ~6.8% of cases harbored a RET fusion [24]. RET fusions are more common in radiation-associated than in “sporadic” cases. Several evidences support the possibility that both internal (to ^131^I and other isotopes) and external irradiation may favor these genomic events [105]. In 1986, large amounts of ^131^I and other isotopes, released by the Chernobyl nuclear power plant, contaminated regions of Belarus, Russia, and Ukraine, thus resulting in a sharp PTC incidence increase in children and adolescents. In one study, ~58% of post-Chernobyl PTCs in patients who were <10 years old at the time of the accident harbored a RET fusion [106]. Prevalence of RET fusions tended to decline after a longer interval from the nuclear accident [16,107]. Moreover, as many as 50% of PTCs in atomic bomb survivors, who were exposed to high radiation doses (>0.5 Gy), harbored RET fusions [108]. Accordingly, RET fusions occur dose-dependently upon irradiation with or X-ray thyrocytes in culture [24,98,109]. 

*RET* fusions have been reported more commonly (and the BRAFV600E mutation more rarely) in pediatric than in adult thyroid cancer patients [16,110,111]. In young patients, the NCOA4-RET fusion occurred more commonly in radiation-associated, particularly in early cases, while CCDC6-RET was more common in “sporadic” cases [112].

RET fusions are uncommon in thyroid cancer subtypes other than PTC [104]. FTC (follicular thyroid carcinoma), the other major type of differentiated thyroid cancer, is generally negative for RET fusions [80]. PDTC (poorly differentiated thyroid carcinoma) and ATC (anaplastic thyroid carcinoma) may derive from pre-existing differentiated carcinomas, including PTC. In the analysis of large databases (more than 60,000 tumor samples), RET fusions were found in 13/560 (2.32%) and 36/500 (7.2 %) PTC cases, 1/107 (0.93%) ATC cases, and 6/134 (4.47%) PDTC cases [18]. Similarly, in a recent study, 5.9% of PDTC but no ATC harbored *RET* rearrangements, suggesting that *RET* fusion-positive PTCs rarely progress to ATC [30]. 

As mentioned above, the most typical oncogenic drivers of MTC, a thyroid carcinoma arising from C-cells, are *RET* point mutations, both in sporadic and familial cases [4,5,6,7,16,17,18]. It is worth noting that RET is normally expressed in C-cells and therefore, its oncogenic conversion does not need the acquisition of a novel transcriptional promoter caused by a gene fusion. However, one MTC case harboring a RET fusion, MYH13-RET, has been recently reported [55].

In terms of the clinical relevance of RET fusions in thyroid cancer, besides the possible therapeutic use of RET TKIs, it should be noted that fine-needle aspiration cytology is commonly used for pre-operative assessment of thyroid nodules, though it is unable to reach a definitive diagnosis in up to 25% of the cases. In these cases, molecular diagnostic methods have been developed to help diagnosis by determining the presence of different oncogenic mutations, including RET fusions [113].

## 6. *RET* Gene Fusions in Non-Small Cell Lung Cancer

RET fusions occur in 1%–2% of lung carcinoma, predominantly in adenocarcinoma (LADC) but also in rare types such as adenosquamous carcinoma [13,17,18,29,50,51,52,114,115,116]. In the same analysis of large databases described above, RET fusions were found in 0.35%–0.88% of LADC (n = 9088) [18]. The most common RET fusions in NSCLC are KIF5B-RET, NCOA4-RET, and CCDC6-RET. KIF5B (Kinesin-1 heavy chain) is by far the most common RET fusion partner in NSCLC, being detected in up to 70%–90% of the cases (Figure 1, Table 1) [17]. The breakpoint cluster region in *KIF5B* may occur in several introns, most commonly in intron 15 [95]. In NSCLC, RET fusions are mutually exclusive with other driver mutations, such as ALK or ROS1 rearrangements or EGFR or KRAS mutations, once again suggesting common signaling mechanisms [13,116].

In LADC, RET fusions are reported to be associated with young age (<60 years), female gender, Asian ethnicity, and minimal tobacco exposure [17]. RET fusions were also associated with poor differentiation, solid subtype, presence of signet ring cells, small tumor size, early lymph node metastases, low levels of PD-L1 expression, and poor response to immunotherapy [13,15,17]. In vitro irradiation with -rays generated KIF5B-RET fusion in lung cells, thus pointing, similar to thyroid cancer, to radiation as a possible risk factor for RET fusion in lung cancer [117]. 

Clinically, there is strong interest in RET TKIs for the targeted treatment of NSCLC [11,12,13,14,15,114,]. Interestingly, RET fusions (CCDC6-RET, NCOA4-RET, and the newly described CDC123-RET) have been reported as an acquired resistance mechanism of LADC to EGFR or ALK tyrosine kinase inhibitors [118,119,120].

## 7. *RET* Gene Fusions in Other Malignancies

In the metanalysis reported by Kohno [18], RET fusions were found in 0.7% of total samples, including cancers other than thyroid and lung ones, such as breast (0.00%–0.21%), colon (0.00%–0.26%), esophageal (0.00%–0.17%), ovarian (0.00%–0.17%), prostate (0.08%), and stomach (0.81%) carcinoma, as well as acute myeloid leukemia (0.00%–0.50%) and very rare cancers such as anaplastic ganglioglioma (a rare CNS tumor of children and young adults), and Erdheim–Chester Disease (a rare form of non-Langerhans cells histiocytosis). In another study, RET fusions were found in 0.6% (27 out of 4871) patients with different malignancies (besides thyroid and lung carcinoma), including ovarian and salivary gland carcinomas [69]. In a further study of nearly 33,000 cases of circulating free tumor DNA (cfDNA) from metastatic patients, *RET* fusion events were identified in NSCLC and in colorectal, breast, and thyroid carcinomas [121]. 

Spitz tumors and Spitzoid melanomas frequently harbor kinase, including RET (3% of cases), fusions [44,56]. Moreover, translocations t(10;22)(q11;q11) and t(6;10)(q27;q11) generating BCR-RET and FGFR1OP-RET fusions have been identified in single cases of chronic myelomonocytic leukemia (CMML) and primary myelofibrosis (PMF) with secondary acute myeloid leukemia (AML) [26,122]. Finally, pediatric spindle mesenchymal tumors are a heterogeneous group of rare soft tissue neoplasms with fibroblastic or neural-like differentiation, that include infantile fibrosarcoma and others [31,36]. These tumors typically feature rearrangements involving ALK, BRAF, NTRK1, NTRK2, NTRK3, and MET kinases [36]. A significant fraction of them has been discovered to harbor RET fusions, including the rare MYH10-, CLIP2-, KIAA1217- SPECC1L-, KHDRBS1-, VCL-, and TFG-RET fusions [13,31,36,54,58,71,75,123]. MYH10-RET fusion, in particular, is, thus far, the most common RET lesion in spindle mesenchymal tumor and it was also identified in a case of infantile myofibromatosis [36,54]. The morphology of RET fusion-positive spindle mesenchymal samples largely overlaps that of NTRK-positive ones [31,36]. 

RET is a transcriptional target of estrogen receptor alpha (ESR1) and several studies have reported its overexpression, particularly in ER+ breast cancer as well as its association with endocrine resistance [124]. In a recent study based on targeted genomic profiling of 9693 cases, RET genomic alterations, including 16 rearrangements, were observed in 1.2% of breast cancers. These rearrangements featured the classical CCDC6-RET and NCOA4-RET fusions, the new uncharacterized RASGEF1A-RET and ZNF485-RET fusions, and rearrangements resulting in tandem duplications that involve exons 12–19 of RET [32]. 

RET fusions have been identified in <1% of colorectal carcinomas (CRC) [33,34,66]. The most common fusions in CRC are CCDC6-RET and NCOA4-RET. More rare fusions involving TNIP1-, SNRNP70-, GEMIN5- and RRBP1 as N-terminal partners were also described (Table 1) [34,35]. In CRC, RET fusions were more frequent in old patients, right-sided, RAS/ BRAF wild-type, and MSI (microsatellite instable)-high tumors and were associated with negative prognosis. Accordingly, as many as 26% of patients with right-sided RAS and BRAF wild-type tumors harbored a RET rearrangement. This fraction significantly increased when only MSI-high CRCs were considered [34]. 

Salivary gland carcinomas are rare and heterogeneous tumors with several different subtypes. Secretory carcinoma, originally described as mammary analogue secretory carcinoma (MASC), is a salivary gland low-grade carcinoma typically associated with ETV6-NTRK3 fusion [42]. Recently, some secretory carcinomas, affecting the parotid or the submandibular gland, and negative for ETV6-NTRK3, were found to harbor ETV6- or NCOA4-RET fusions [42]. Recurrent rearrangements involving the RET gene were also identified in a subset of intraductal carcinoma (IC), another salivary duct carcinoma that shares some morphologic and immunophenotypical features with secretory carcinoma. Specifically, more than 40% of the IC cases harbored RET fusions, including NCOA4-RET, TRIM27-RET, and KIAA1217-RET [59,60,125].

## 8. Conclusions

Though initially believed to be PTC-specific, *RET* gene fusions have more recently been identified in several cancer types, including lung, colon, and breast carcinoma. In terms of clinical translation, this has significantly raised the interest for *RET* as far as the possibility of developing molecular diagnostic methods as well as targeted treatments with RET TKIs. On the other hand, from a mechanistic point-of-view, RET fusion was revealed to be an interesting model to study the mechanism of gene rearrangement and the role of radiation in cancer.

## Figures and Tables

**Table 1:** *RET* (REarranged during Transfection) gene fusions in human neoplasms.

Fusion ^1^ (Alternative Name)	Features of the 5’-Terminal Fusion Partner			Neoplasm ^5^	Reference
	Alternative Name	Protein Domains ^4^	Chr. Location		
ACBD5-RET	ACBD5	ABCP (1), TM (2), CC	10p12.1	PTC	[22]
AFAP1L2-RET	AFAP1L2 (XB130)	PH (3), CC	10q25.3	PTC	[23]
AKAP13-RET	AKAP13	R II binding (4), Rho GEF (5), PH (3), CC	15q25.3	PTC	[24]
ANKRD26-RET	ANKRD26	ANK (6), CCDC144C (7), CC	10p12.1	PTC	[25]
BCR-RET	BCR	Bcr-Abl oligo (8), Rho GEF (5), C2 (9), Rho GAP (10), TM (2), CC	22q11.23	CMML	[26]
CDC123-RET	CDC123	D123 (11)	10p14-p13	LADC	[27]
CCDC6-RET (RET/PTC1)	CCDC6 (H4)	DUF (12), CC	10q21.2	PTC, PDTC, LADC, BRCA, SCT, CRC, STAD	[21,28,29,30,31,32,33,34,35]
CLIP2-RET	CLIP2	CAP-Gly (13), CC	7q11.23	SCT, LPF	[31,36]
CUX1-RET	CUX1	CUT:CUT (14), Homeobox (15), CC	7q22.1	LADC	[37]
DLG5-RET	DLG5	Takusan (16), PDZ (17), dbPDZ (18), GuKc (19), CC	10q22.3	PTC	[38]
EPHA5-RET	EPHA5	-	4q13.1/2	LADC	[39]
ERC1-RET	ERC1 (ELKS)	Cast (20), RBD FIP (21), CC	12p13.33	PTC, BRCA	[38,40,41]
ETL4-RET	KIAA1217	AIP (22), CC	10p12.1/2	SCT, LPF, IC	[36]
ETV6-RET	ETV6 (TEL)	SAM PNT (23), ETS (24)	12p13.2	SC	[42]
FGFR1OP-RET	FGFR1OP (FOP)	FOP dimer (25)	6q27	CMML, PMF	[26]
FKBP15-RET	FKBP15	FKBP (26), CC (74)	9q32	PTC	[24]
FRMD4A-RET	FRMD4A	FERM (27), DEF (12), CC	10p13	LADC	[43]
GEMIN5-RET	GEMIN5	WD40 (28), ANAPC4 WD40 (29), CC	5q33.2	CRC	[35]
GOLGA5-RET (RET/PTC5)	GOLGA5 (RFG5)	Golgin A5 (30), TM (2), CC	14q32.12	PTC, SN	[44,45]
HOOK3-RET	HOOK3	HOOK (31), CC	8p11.21	PTC	[46]
KHDRBS1-RET	KHDRBS1 (Sam68)	-	1p35.2	IFS-like	[36]
KIAA1468-RET	KIAA1468 (RELCH)	LisH (32), HEAT (33), CC	18q21.33	IMA, PTC	[25,47,48]
KIF13A-RET	KIF13A	Kinesin (34), Kinesis-as (35), FHA (36), KIF1B (37) GuKc (19), CC	6p22.3	LADC	[49]
KIF5B-RET	KIF5B	Kinesin (34), CC	10p11.22	LADC, AS, SN	[29,44,50,51,52]
KTN1-RET (RET/PTC8)	KTN1 (RFG8)	CC	14q22.3	PTC	[53]
MYH10-RET	MYH10	Myosin N (38), Myosin head (39), IQ (40), Myosin tail (41), CC	17p13.1	IM, SCT	[31,36,54]
MYH13-RET	MYH13	Myosin N (38), Myosin head (39), Myosin tail (41), CC	17p13.1	MTC	[55]
MYO5A-RET	MYO5A	Myosin head (39), IQ (40), DUF (12), CC (	15q21.2	PSCN	[56]
MYO5C-RET	MYO5C	Myosin head (39), IQ (40), TM (2), DUF (12), CC	15q21.2	LADC	[57]
NCOA4-RET (RET/PTC3)	NCOA4 (RFG, ELE1, ARA70)	ARA70 (42), CC	10q11.22	PTC, PDTC, LADC, BRCA, IC, SCT, CRC	[27,30,31,32,33,34,35,52,58,59,60,61,62,63]
PCM1-RET	PCM1	PCM1 C (43), CC	8p22	PTC	[64]
PDCD10-RET	PDCD10	-	3q26.1	PDTC	[21]
PICALM-RET	PICALM	-	11q14.2	LADC	[39]
PPFIBP2-RET	PPFIBP2	SAM PNT (23), CC	11p15.4	PTC	[23]
PRKAR1A-RET (RET/PTC2)	PRKAR1A	RIIa (44), cNMP (45), CC	17q24.2	PTC	[65]
RASGEF1A-RET (ΔRET)^2^	RASGEF1A	RAS GEF N (46), RAS GEF (47), CC	10q11.21	BRCA	[32]
RASSF4-RET	RASSF4	RA (48), Nore1-SARAH (49), CC	10q11.21	LADC	[21]
RET-RET ^3^	RET	CLD1-4 (50), TM (2), TK (51)	10q11.21	BRCA	[32]
RRBP1-RET	RRBP1	TM (2), Rib recp KP reg (52), CC	20p12.1	CRC	[66]
RUFY2-RET	RUFY2	RUN (53), FYVE (54), CC	10q21.3	LADC, PTC	[67,68]
SNRNP70-RET	SNRNP70	U1snRNP70 N (55), RRM 1 (56), CC	19q13.33	CRC	[34]
SPECC1L-RET	SPECC1L	CH (57), CC	22q11.23	PTC, LPF, LPF-NT	[24,31]
SQSTM1-RET	SQSTM1	-	5q35.3	PTC	[69]
TBC1D32-RET	TBC1D32	BROMI (58), TM (2), CC	6q22.31	LADC	[70]
TBL1XR1-RET	TBL1XR	LisH (32), WD40 (28)	3q26.32	PTC	[24]
TFG-RET	TFG	PB1 (59), CC	3q12.2	PDTC, SCT, LPF	[21,71]
TNIP1-RET	TNIP1	UBD (60), CC	5q33.1	CRC	[34]
TRIM24-RET (RET/PTC6)	TRIM24	zf-B box (61), PHD (62), Bromodomain (63), CC	7q32-34	PTC, LADC, CRC	[34,67,72]
TRIM27-RET	TRIM27 (RFP)	zf-C3HC4 4 (64), zf-B box (61), PRY (65), SPRY (66), CC	6p22.1	PTC, IC	[24,59,60]
TRIM33-RET (RET/PTC7)	TRIM33	zf-RING UBOX (67), zf-B box (61), PHD finger (62), Bromodomain (63), CC	1p13.2	PTC, LADC	[72,73]
UEVLD-RET	UEVLD	UEV (68), LDH (69)	11p15.1	PTC	[74]
VCL-RET	VCL	VINC (70), CC	10q22.2	LPF	[75]
WAC-RET	WAC	WW (71), CC	10p12.1	LADC	[76]
ZNF485-RET	ZNF485	KRAB (72), ZF-C2H2 (73)	10q11.21	BRCA	[32]

^1^ Some RET fusions (EPHA5-RET, KHDRBS1-RET, PICALM-RET, SQSTM1-RET) are listed here but not reported in Figure 1 because of the lack of information about the involved exon. Moreover, additional RET fusion partners have been recently listed but without molecular details: CLIP1 and PRKG1 (15) and EML4 and PARD3 (14). ^2^ The involved RASGEF1A (Rat Sarcoma Guanine nucleotide Exchange Factor) portion (5’-UTR) is non-coding; thus, this fusion generates a truncated RET (ΔRET) protein starting at a cryptic ATG site in RET exon 11. ^3^ These rearrangements result in tandem duplications fusing one copy of RET (exons 1–20) to a second copy of RET starting from exon 7, 9, or 12. ^4^ Protein domains legend (PFAM, https://pfam.xfam.org/) (domains are numbered as in Figure 1): (1) ACBP: Acyl CoA binding protein; (2) TM: transmembrane domain; (3) PH: Actin filament associated protein family Pleckstrin homology (PH) domain; (4) RII: RII binding domain; (5) RhoGEF: Guanine nucleotide exchange factor for Rho/Rac/Cdc42-like GTPases; (6) ANK: ankyrin repeat domain; (7) CCDC144C: protein coiled region; (8) Bcr-Abl oligo Bcr-Abl oligomerization domain; (9) C2: C2 domain; (10) Rho GAP: GTPase activator proteins towards Rho/Rac/Cdc42-like small GTPases; (11) D123; (12) DUF: Domain of unknown function; (13) CAP-Gly: Glycine-rich domain of cytoskeleton-associated proteins (CAPs); (14) CUT:CUT (also known as ONECUT domain), DNA-binding motif; (15) Homeobox: homeobox domain or homeodomain; (16) Takusan: in Japanese ’many’, found in protein regulating synaptic activity; (17) PDZ: Post synaptic density protein, Drosophila disc large tumor suppressor, and zonula occludens-1 protein domain; (18) dbPDZ: PDZ-associated domain; (19) GuKc: Guanylate kinase homology domain; (20) Cast: RIM-binding protein of the cytomatrix active zone; (21) RBD-FIP: Rab11-binding domain (RBD) at the C-terminus of a family of Rab11-interacting proteins (FIPs); (22) AIP: Actin-interacting protein; (23) SAM PNT: Sterile alpha motif (SAM)/Pointed domain; (24) ETS: erythroblast transformation specific domain; (25) FOP dimer: Fibroblast growth factor receptor 1 (FGFR1) oncogene partner (FOP) N terminal dimerization domain; (26) FKBP: FKBP-type peptidyl-prolyl cis-trans isomerase domain; (27) FERM: FERM domain (4.1 protein, Ezrin, Radixin and Moesin); (28) WD40: WD40 repeat (also known as the WD or beta-transducin repeat); (29) ANAPC WD40: Anaphase-promoting complex subunit 4 WD40 domain; (30) Golgin A5: Golgin subfamily A member 5 domain; (31) HOOK: HOOK domain; (32) LisH: Lissencephaly type-1-like homology motif; (33) HEAT: HEAT repeat tandem repeat structural motif (Huntingtin, Elongation factor 3, Phosphatase 2A, and the yeast kinase TOR1); (34) Kinesin: Kinesin motor domain; (35) Kinesin-as: Kinesin-associated domain; (36) FHA: Forkhead associated domain; (37) KIF1B: Kinesin protein 1B domain; (38) Myosin N: Myosin N-terminal SH3-like domain; (39) Myosin head: motor domain; (40) IQ: IQ calmodulin-binding motif; (41) Myosin tail: Myosin tail domain; (42) ARA70 (Androgen Receptor Activator 70): Nuclear Coactivator domain; (43) PCM1 C: Pericentriolar material 1 C terminus domain; (44) RIIa: Regulatory subunit of type II PKA R-subunit; (45) cNMP: Cyclic nucleotide-binding domain; (46) RAS GEF N: RAS GEF (Rat Sarcoma Guanine nucleotide Exchange Factor) N-terminal motif; (47) RAS GEF: RAS GEF (Rat Sarcoma Guanine nucleotide Exchange Factor) domain; (48) RA: Ras association (RalGDS/AF-6) domain; (49) Nore1-SARAH: Novel Ras effector 1 C-terminal SARAH (Sav/Rassf/Hpo) domain; (50) CLD1-4: RET Cadherin-like domain 1–4; (51) TK: Tyrosine Kinase domain; (52) Rib recp KP reg: Ribosome receptor lysine/proline-rich region; (53) RUN: RUN domain; (54) FYVE: FYVE zinc finger domain; (55) U1snRNP70 N: U1 small nuclear ribonucleoprotein of 70 kDa MW N terminal domain; (56) RRM 1: RNA recognition motif; (57) CH: Calponin homology domain; (58) BROMI: Broad-minded protein; (59) PB1: Phox and Bem1 domain; (60) UBD: Ubiquitin-binding domain; (61) zf-B box: B-box-type zinc finger domain; (62) PDH: Plant Homeo Domain; (63) Bromodomain; (64) zf-C3HC4 4: Zinc finger of C3HC4-type, RING; (65) PRY: SPRY-associated domain; (66) SPRY: Spla/ryanodine receptor domain and socs box-containing 3 domain; (67) zf-RING UBOX: RING-type zinc-finger domain; (68) UEV: N-terminal ubiquitin E2 variant domain; (69) LDH: lactate/malate dehydrogenase, NAD-binding domain; (70) VINC: Viculin Family domain; (71) WW: rsp5-domain or WWP repeating motif; (72) KRAB: Kruppel-associated box; (73) ZF-C2H2: zinc finger; (74) CC: coiled-coil. ^5^ AS (lung adenosquamous cell carcinoma), BRCA (breast invasive carcinoma), CMML (Chronic myelomonocytic leukaemia), CRC (colorectal carcinoma), IC (intraductal carcinoma of the salivary gland), IFS (infantile fibrosarcoma), IM (infantile myofibromatosis), IMA (invasive mucinous lung adenocarcinoma), LADC (lung adenocarcinoma), LPF (lipofibromatosis), LPF-NT (lipofibromatosis-like neuronal tumors), MTC (medullary thyroid carcinoma), PDTC (poorly differentiated thyroid carcinoma), PMF (primary myelofibrosis), PTC (papillary thyroid carcinoma), PSCN (pigmented spindle cell nevus of Reed), SC (secretory carcinoma of the salivary gland), SCT (spindle cell tumor of soft tissues), SN (spitzoid neoplasms), STAD (stomach adenocarcinoma).
